# Reducing hypoxia and inflammation during invasive pulmonary aspergillosis by targeting the Interleukin-1 receptor

**DOI:** 10.1038/srep26490

**Published:** 2016-05-24

**Authors:** Mark S. Gresnigt, Abdessalem Rekiki, Orhan Rasid, Amélie Savers, Grégory Jouvion, Eric Dannaoui, Marianna Parlato, Catherine Fitting, Matthias Brock, Jean-Marc Cavaillon, Frank L. van de Veerdonk, Oumaïma Ibrahim-Granet

**Affiliations:** 1 Unité de recherche Cytokines & Inflammation, Institut Pasteur, Paris; 2 Department of Medicine, Radboud University Medical Center, Nijmegen, The Netherlands; 3 PFID, Bioaster, Institut Pasteur, Paris France; 4 Fungal Genetics and Biology, School of Life Sciences, University of Nottingham, UK; 5 Unité Histopathologie Humaine et Modèles Animaux, Institut Pasteur, Paris France; 6 Paris-Descartes University, Faculty of Medicine, APHP, European Georges Pompidou Hospital, Parasitology-Mycology Unit, Microbiology department, Paris, France; 7 INSERM UMR S1163 Institut Imagine, Laboratoire d’Immunité Intestinale, Paris France

## Abstract

Hypoxia as a result of pulmonary tissue damage due to unresolved inflammation during invasive pulmonary aspergillosis (IPA) is associated with a poor outcome. *Aspergillus fumigatus* can exploit the hypoxic microenvironment in the lung, but the inflammatory response required for fungal clearance can become severely disregulated as a result of hypoxia. Since severe inflammation can be detrimental to the host, we investigated whether targeting the interleukin IL-1 pathway could reduce inflammation and tissue hypoxia, improving the outcome of IPA. The interplay between hypoxia and inflammation was investigated by *in vivo* imaging of hypoxia and measurement of cytokines in the lungs in a model of corticosteroid immunocompromised and in *Cxcr2* deficient mice. Severe hypoxia was observed following *Aspergillus* infection in both models and correlated with development of pulmonary inflammation and expression of hypoxia specific transcripts. Treatment with IL-1 receptor antagonist reduced hypoxia and slightly, but significantly reduced mortality in immunosuppressed mice, but was unable to reduce hypoxia in *Cxcr2*^−/−^ mice. Our data provides evidence that the inflammatory response during invasive pulmonary aspergillosis, and in particular the IL-1 axis, drives the development of hypoxia. Targeting the inflammatory IL-1 response could be used as a potential immunomodulatory therapy to improve the outcome of aspergillosis.

Humans continuously inhale spores of the fungus *Aspergillus fumigatus*, which is a ubiquitous mould in soil and decaying organic debris. Although rarely causing disease in immunocompetent individuals, *A. fumigatus* can cause lethal invasive pulmonary aspergillosis (IPA) in immunocompromised patients, with mortality varying between 30% and 90%^1^. Therapies that result in neutropenia or neutrophil dysfunction are major predisposing factors^2^,^3^,^4^. In addition, patients that receive high doses of corticosteroids display an increased susceptibility to IPA^3^,^4^,^5^.

Pulmonary hypoxia is commonly observed during experimental *in vivo* pulmonary aspergillosis models^6^,^7^,^8^ and patients with IPA^9^, and is associated with poor outcome. It is believed that destruction of the pulmonary tissue by fungal invasion^6^ and inhibition of angiogenesis^10^ stands at the basis of pulmonary hypoxia in aspergillosis. In addition, collateral damage as a result of the inflammatory response contributes to hypoxia during aspergillosis^6^,^7^. An important evolutionary adaptation of *A. fumigatus* has been to survive and proliferate in hypoxic environments, this is further illustrated by the fact that most *Aspergillus* mutants that are incapable of adapting to hypoxic conditions are avirulent or less virulent in mouse models^7^,^11^,^12^,^13^,^14^,^15^. It has even been suggested that the hypoxic microenvironment during aspergillosis contributes to fungal invasion^8^.

Although the inflammatory response is required for tissue repair and clearance of *A. fumigatus*, unresolved chronic inflammation that occurs when *Aspergillus* spores are not cleared or restricted in germination can be detrimental to the host^16^,^17^. Hypoxia caused by tissue damage and ischemia will trigger necrotic cell death and the release of danger signals, such as interleukin (IL-1α)^18^, which drives the inflammatory response. The inflammatory response as a result of hypoxic cell death leads to the recruitment of immune cells, activation of downstream signalling pathways, and induction of proinflammatory cytokines and chemokines^19^,^20^. The ability of human macrophages to produce IL-1 and tumour necrosis factor (TNFα) responses is enhanced in hypoxic environments^21^. Pulmonary hypoxia and inflammation intertwine at the cellular and molecular level^22^ with hypoxia being able to augment inflammation which in turn contributes to development of inflammation at the site of infection.

Here we studied hypoxia and its correlation with inflammation in two murine models of invasive pulmonary aspergillosis. First, *Cxcr2*^−/−^ mice that are not able to efficiently recruit neutrophils to the site of infection were studied^23^,^24^. Second we investigated mice treated with cortisone-acetate that simulates corticosteroid immunosuppression in patients and make them susceptible to aspergillosis. Our approach focused on *in vivo* real-time monitoring of hypoxia through the HS680 probe which was used previously to measure tumor hypoxia^25^. The goal of the current studies was to use HS680 for detecting up-regulation of carbonic anhydrase (CAIX) *in vivo* and to validate the specific accumulation of the agent in hypoxic regions of lungs tissues using *in vivo* imaging and quantification of hypoxia by fluorescence molecular tomography and by gene expression. After correlating hypoxia to cytokine mediated inflammation in the lung, we further investigated whether local tissue hypoxia could be reduced by targeting the IL-1 pathway that could be a driving force in the hypoxia- inflammatory loop during IPA.

## Results

### Cxcr2^−/−^ mice develop severe hypoxia and inflammation during invasive pulmonary aspergillosis

Neutrophils are, in addition to alveolar macrophages, essential for clearing *Aspergillus* spores from the lungs^6^,^23^,^24^,^26^. We investigated the role of CXCR2-mediated neutrophil recruitment in the development of hypoxia and inflammation during pulmonary aspergillosis by comparing *Cxcr2*^−/−^ mice with wild-type mice.

**Figure 1:**
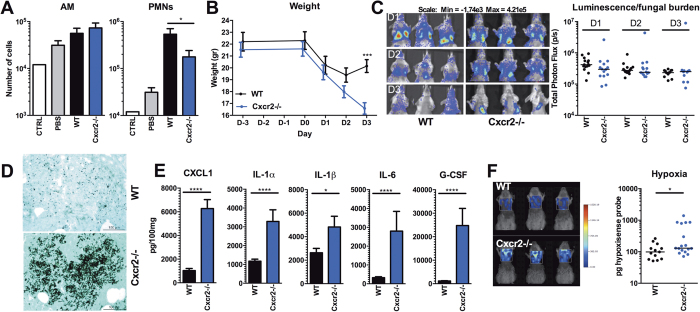
Hypoxia and inflammation during aspergillosis in *Cxcr2*^−/−^ mice (**A**) Number of Alveolar macrophages and neutrophils (PMNs) in the BAL of control (naïve), PBS treated, wild-type (WT) and *Cxcr2*^−/−^ mice 1 day p.i with live resting *Aspergillus fumigatus* spores (5 × 107 in 25 μl), shown as scatterplot and median and compared for significance using Mann Whitney U test. (**B**) Weight of WT (n = 13); and *Cxcr2*^−/−^ mice (n = 13) during the course of infection and compared for significance by two way ANOVA. (**C**) Fungal burden measured by the luminescence signal by *in vivo* conversion of luciferin by the luciferase expressing *A. fumigatus* (strain 2/7/1) at day 1, 2 and 3 p.i. (**D**) Representative lung sections from WT and *Cxcr2*^−/−^ mice at day 3 p.i. Methenamine silver staining shows, non-germinating conidia in WT and, foci of germinating conidia in *Cxcr2*^−/−^ mice. (**E**) Proinflammatory mediators CXCL1, IL-1α, IL-1β, IL-6, and G-CSF measured in lung homogenates at day 3 p.i in WT and *Cxcr2*^−/−^ mice, and are represented as mean ± SEM and were compared for significance using the Mann-Whitney U test. (**F**) Hypoxia measured by the fluorescent HypoxiSense probe 680 (Perkin Elmer) in the lungs of WT and *Cxcr2*^−/−^ mice at day 3 pi. Data is represented as scatterplot and median and were compared for significance using the Mann-Whitney U test. *p < 0.05;**p < 0.005; ***p < 0.001.

*Cxcr2*^−/−^ mice and WT controls were intranasally infected with a dose of 5 × 10^7^ live dormant conidia. At day 2 post infection (p.i) the number of alveolar macrophages (AM) and neutrophils (PMNs) was evaluated in the bronchoalveolar lavage (BAL). Compared to wild-type mice, *Cxcr2*^−/−^ mice did not show a difference in absolute number of alveolar macrophages (Fig. 1A). In contrast and in line with previous studies^23^,^24^, *Cxcr2*^−/−^ mice demonstrated an impaired recruitment of neutrophils to the lungs following *Aspergillus* infection, which was twofold lower than the amount of neutrophils recruited to the lungs of WT mice (Fig. 1A). During the course of infection, both WT mice and *Cxcr2*^−/−^ mice decrease in weight. While WT mice start to recover from day 3 p.i *Cxcr2*^−/−^ continue to decrease in weight, and the average weight loss of the knock-out (KO) mice is significantly greater than that of WT mice (Fig. 1B) indicating their increased susceptibility to infection. The fungal burden was measured by *in vivo* bioluminescence imaging of the luciferase expressing *A. fumigatus* strain (Fig. 1C). The bioluminescence assay indicates that *Cxcr2*^−/−^ mice do not have a different fungal burden than wild-type animals. It must be noted, however, that under extreme hypoxic conditions the luminescence might be biased towards indicating a lower fungal burden, since the firefly luciferase luminescence signal is oxygen dependent^6^,^27^. To control this possible limitation, lung sections were examined for fungal burden using Grocott methamine silver staining (Fig. 1D). Histological analysis revealed hyphae concentrated in inflammatory foci in the lungs of *Cxcr2*^−/−^ mice, while in WT lungs no hyphal invasion was observed and only non germinating conidia were found.

Inflammation in the lungs was assessed by determining cytokine levels in lung homogenates at day 3 pi. Compared to wild-type mice, *Cxcr2*^−/−^ mice had significantly higher inflammation in the lungs, as demonstrated by higher levels of all measured inflammatory mediators in the lung homogenates (Fig. 1E). Hypoxia in the lungs was assessed at day 3 p.i. by following the activity of carbonic anhydrase using the Perkin Elmer hypoxiSense 680 probe. The hyper-inflammatory state in the lungs of *Cxcr2*^−/−^ mice correlated with significantly elevated hypoxia (Fig. 1F).

### Cortisone-acetate immunocompromised mice demonstrate severe hypoxia and inflammation

Acquired immunodeficiency due to immunosuppressive therapy is one of the major predisposing factors for IPA^2^,^3^,^4^,^5^. Therefore, we investigated the development of hypoxia and inflammation in the setting of corticosteroid immunosuppression. In this setting immunocompetent as well as immunosuppressed mice received an inoculum of 5 × 10^5^ conidia. A higher concentration of 5 × 10^7^ results in the death of immunosuppressed mice within 24 to 48h (data not shown).

**Figure 2:**
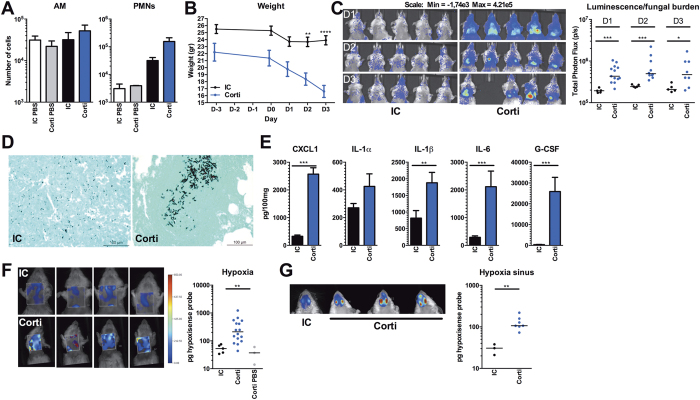
Hypoxia and inflammation during aspergillosis in corticosteroid immunosuppressed mice (**A**) Number of Alveolar macrophages and neutrophils (PMNs) in the BAL of control, PBS treated and *Aspergillus*-infected (5 × 10^5^) immunocompetent (IC n = 5) and cortisone-acetate immunosuppressed mice (Corti n = 13) mice 1 day p.i, shown as scatterplot and median. (**B**) Weight of IC and cortisone-acetate immunosuppressed mice during the course of infection, and compared for significance using a two way ANOVA. (**C**) Fungal burden measured by the luminescence signal by *in vivo* conversion of luciferin by the luciferase expressing *A. fumigatus* (strain 2/7/1) at day 1, 2 and 3 p.i. Data is represented as scatterplot and median and were compared for significance using the Mann-Whitney U test. (**D**) Representative lung sections from (IC) and (Corti) mice at day 3 p.i. Methenamine silver staining showing, non-germinating conidia in (IC) and foci of germinating conidia in (Corti) mice. (**E**) Proinflammatory mediators CXCL1, IL-1α, IL-1β, IL-6, and G-CSF measured in lung homogenates at day 3 p.i in IC and cortisone-acetate immunosuppressed mice, and are represented as mean ± SEM and compared for significance using the Mann-Whitney U test. (**F**) Hypoxia measured by the fluorescent HypoxiSense 680 probe (Perkin Elmer) in the lungs of IC and Corti mice at day 3 p.i, and shown as scatterplot with median and were compared for significance using the Mann-Whitney U test. (**G**) Hypoxia measured in the sinus of IC and Corti mice at day 3 p.i, and shown as scatterplot with median and were compared for significance using the Mann-Whitney U test. *p < 0.05;**p < 0.005; ***p < 0.001.

First, the effect of cortisone-acetate immunosuppression on the recruitment of alveolar macrophages and neutrophils to the lungs was investigated by measuring AMs and PMNs in the BAL at 2 days p.i. Following infection, no significant change in the recruitment of both cell subsets was observed between immunocompetent and cortisone acetate-treated mice (Fig. 2A). Yet it must be noted that corticosteroids affect effector cell function and lifespan, thereby compromising the mice rather than altering cell influx. During the course of infection, the average weight loss of immunosuppressed mice was significantly higher than that of immunocompetent mice (Fig. 2B). Bioluminescence as well as lung sections revealed higher fungal burden with significantly higher luminescent signal and growing hyphae concentrated in inflammatory foci in the lungs of the immunosuppressed mice. As expected, immunocompetent mice showed low luminescence signal and no germination of conidia (Fig. 2C,D). Three days after *Aspergillus* infection, cortisone-acetate immunosuppressed mice had severe pulmonary inflammation, reflected by elevated levels of CXCL1, IL-1α, IL-1β, IL-6, and G-CSF compared to immunocompetent mice (Fig. 2E). In correlation with severe pulmonary inflammation, cortisone-acetate immunosuppressed mice demonstrated severe pulmonary hypoxia (Fig. 2F) in comparison to immunocompetent and immunosuppressed PBS-infected mice. In addition to pulmonary hypoxia, hypoxia was also significantly higher in the sinus area of immunosuppressed infected mice (Fig. 2G).

### Blockade of IL-1 signalling reduces pulmonary hypoxia in cortisone-acetate immunosuppressed mice

Although crucial for initiating the primary host defence mechanisms against *Aspergillus*^28^,^29^, IL-1 can also play a key role in inducing detrimental hyper-inflammation^17^ and activates hypoxia-inducible factor 1 alpha (Hif1α)^30^,^31^. In turn Hif1α can activate transcription of IL-1α and IL-1β^32^,^33^. We therefore hypothesized that inhibition of IL-1 signalling, by IL-1Receptor antagonist (Ra) (Anakinra), could break the loop between inflammation and hypoxia. IL-1Ra reduces the biological activity of IL-1 by competitively binding to the Interleukin-1 type 1 receptor and can be therapeutically used to target IL-1 mediated inflammation^34^. Anakinra is the recombinant form of human IL-1Ra but has been shown to be active in murine models^35^,^36^.

**Figure 3:**
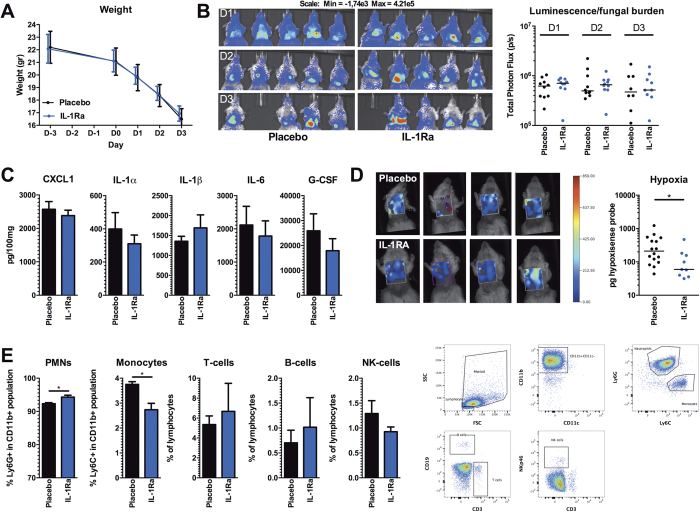
IL-1Ra (Anakinra) treatment reduces hypoxia in corticosteroid immunosuppressed mice (**A**) Weight of placebo (n = 16) and IL-1Ra (n = 10) treated cortisone-acetate immunosuppressed mice during the course of infection. (**B**) Fungal burden measured by the luminescence signal by *in vivo* conversion of luciferin by the luciferase expressing *A. fumigatus* (strain 2/7/1) at day 1, 2 and 3 p.i. Data is represented as scatterplot and median and were compared for significance using the Mann-Whitney U test. (**C**) Pro-inflammatory mediators CXCL1, IL-1α, IL-1β, IL-6, and G-CSF measured in lung homogenates at day 3 p.i in placebo and IL-1Ra-treated cortisone-acetate immunosuppressed mice, and are represented as mean ± SEM and were compared for significance using the Mann-Whitney U test. (**D**) Hypoxia measured by the HypoxiSense 680 probe (Perkin Elmer) at day 3 p.i, and is shown as scatterplot and median and was compared for significance using the Mann-Whitney U test. *p < 0.05. (E) Percentages of Neutrophils (CD11b^+^Ly6G^high^Ly6C^mid^), Monocytes (CD11b^+^Ly6C^high^Ly6G^mid^), T cells (CD3^+^), B cells (CD19^+^), and NK cells (NKp46^+^CD3^−^) (graphs) and representative gating strategy (right plots) at day 3 p.i. in placebo and IL-1Ra-treated cortisone-acetate immunosuppressed mice. Data in graphs are presented as mean ± SEM and were compared for significance using the Mann-Whitney U test. *p < 0.05.

Cortisone acetate immunosuppressed mice were infected with *Aspergillus* and IL-1Ra treatment was initiated one day p.i, to allow an initial immune response to clear fungal spores. The IL-1Ra-treated group and placebo group demonstrated similar weight loss during the course of infection (Fig. 3A). In addition, fungal burden shown by the luminescence in the lungs of IL-1Ra-treated mice was not significantly different from placebo treated animals (Fig. 3B). Some mice (n = 6) clearly demonstrated the capacity of IL-1Ra to significantly reduce pulmonary inflammation compared to the control group. However, this was not consistently seen in all mice (total: n = 10) and no significant difference in pulmonary inflammation between IL-1Ra-treated, and placebo-treated mice at day 3 p.i could be demonstrated (Fig. 3C). However, IL-1Ra-treatment was effective since hypoxia measured by carbonic anhydrase activity was significantly decreased under this treatment (Fig. 3D).

To explore the effect of IL-1Ra treatment on the cellular composition of the leukocytes that are present in the lung, flow cytometry of lung homogenates was performed at day 3 p.i. IL-1Ra-treated mice show a minor increase in the population Ly6G^high^ neutrophils, whereas the population of Ly6C^high^ monocytes among CD11b^+^ cells in the lungs was reduced (Fig. 3E).

### IL-1Ra treatment reduces expression of hypoxia inducible transcripts

**Figure 4:**
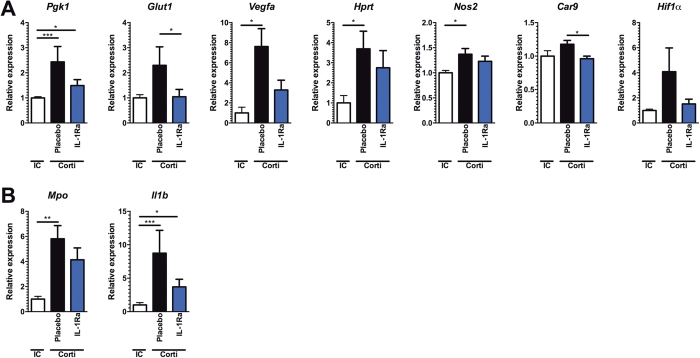
IL-1Ra (Anakinra) treatment reduces expression of hypoxia inducible transcripts. (**A**–**C**) mRNA expression in lung homogenates at day 3 p.i of *Aspergillus*-infected immunocompetent (IC) and corticosteroid immune suppressed mice (Corti) mice, that were either placebo treated or treated with IL-1Ra (Anakinra). mRNA expression of (**A**) the hypoxia inducible genes *Pgk1, Glut1, Vegfa, Hprt, Nos2, Car9* (**B**) their transcription factor *Hif1α* and (**C**) inflammatory genes *Mpo* and *Il1b* was assessed and normalized against housekeeping gene *Gapdh*. The expression is relative to the expression in the immunocompetent group which was set at 1 ± SEM. Data is represented as mean ± SEM and were compared for significance using the Mann-Whitney U test. *p < 0.05; **p < 0.005; ***p < 0.001.

To validate that IL-1Ra treatment of cortisone acetate immunosuppressed mice reduces pulmonary hypoxia during aspergillosis, the effect on expression of hypoxia inducible genes was assessed. Numerous genes are regulated by hypoxia responsive elements (HRE) that are targeted by the transcription factors Hif1α and Hif2α^37^. The expression of several hypoxia inducible genes was assessed in lung homogenates of *Aspergillus*-infected immunocompetent mice and cortisone acetate immunosuppressed mice, with or without IL-1Ra treatment. Compared to immunocompetent mice immunosuppressed mice have significantly elevated expression of the hypoxia inducible genes *Pgk1, Vegfa, Hprt* and *Nos2.* In addition a trend towards increased expression of *Glut1* and *Hif1α* itself could be observed. In contrast, immunocompromised mice that were treated with IL-1Ra did not as strongly induce the hypoxia inducible genes, and some genes *Glut1* and *Car9* (CAIX) were even significantly lower compared to placebo treated mice (Fig. 4A). In addition, expression of *Il1b* and *Mpo* were assessed as markers of inflammation. Both genes were significantly higher expressed in immunosuppressed mice, yet only a mild reduction could be observed following IL-1Ra treatment (Fig. 4B).

### Blockade of IL-1 signalling decreases lethal outcome of aspergillosis in corticosteroid-treated mice

**Figure 5:**
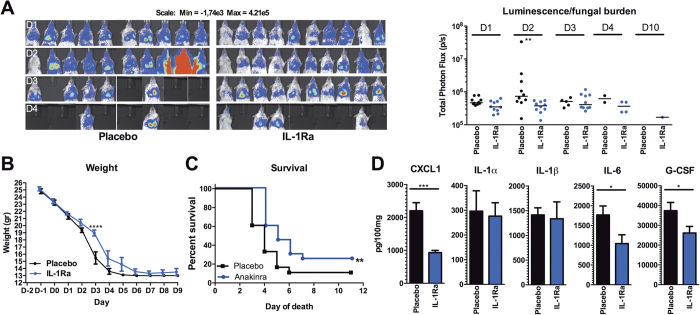
IL-1Ra (Anakinra) treatment reduces weight loss and mortality of corticosteroid immunosuppressed mice (**A**) Fungal burden measured by the luminescence signal by *in vivo* conversion of luciferin by the luciferase expressing *A. fumigatus* (strain 2/7/1) at day 1 to 4 and 10 p.i. Shown as scatterplot and median, means were compared for significance using the Mann Whitney U test. (**B**) Weight of placebo (n = 10) and Anakinra (n = 10) treated cortisone-acetate immunosuppressed mice during the course of infection. Groups were compared using a two way ANOVA. (**C**) Kaplan Meier curve of survival of placebo versus IL-1Ra treated cortisone-acetate immunosuppressed mice. (**D**) Pro-inflammatory mediators CXCL1, IL-1α, IL-1β, IL-6, and G-CSF measured in lung homogenates at the day of death in placebo and IL-1Ra treated cortisone-acetate immunosuppressed mice. Data is represented as mean ± SEM and were compared for significance using the Mann-Whitney U test. *p < 0.05; **p < 0.005; ***p < 0.001.

We further explored whether IL-1 receptor blockade, which results in reduction of inflammation and hypoxia, could increase survival in cortisone-acetate immunosuppressed mice. In mice that were infected with 5 × 10^5^ conidia no significant effect of IL-1Ra treatment on survival of the mice could be observed when compared to PBS treatment (data not shown). However, when the dose was reduced to 2 × 10^5^, treatment with IL-1Ra resulted in a significant reduction of fungal burden as shown by bioluminescence signal (Fig. 5A) associated with a significant difference in the body weights at day 3 p.i (Fig. 5B). Treatment with IL-1Ra for 10 days improved the survival of mice from 11 to 25% (Fig. 5C). Assessment of inflammation in the lungs at the day of death demonstrated that IL-1Ra treated mice had significantly lower levels of IL-6, CXCL1, and G-CSF in their lungs compared to placebo treated mice at the time of death (Fig. 5D).

In a real patient situation antifungal treatment is the standard regimen and immunomodulatory therapy could be added to the standard care. There is a possibility that immunomodulatory therapy could be more beneficial in combination with antifungal strategies. Therefore a pilot experiment comparing the survival of immunosuppressed mice challenged *Aspergillus-*conidia (2 × 10^5^) treated either with Caspofungin (10 mg/kg/day) or with a combination of Caspofungin and IL-1Ra was performed. Interestingly, the combination was well tolerated by the mice and addition of IL-1Ra to the treatment regimen improved survival from 40% with caspofungin to 60% (Fig. S1).

### Blockade of IL-1 signalling does not influence hypoxia and inflammation in *Cxcr2* ^−/−^ mice

**Figure 6:**
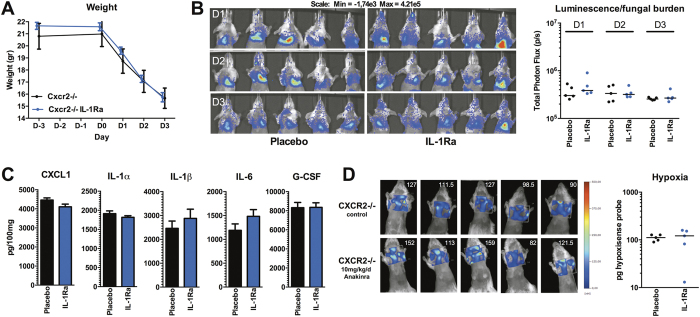
IL-1Ra (Anakinra) treatment does not reduce hypoxia and inflammation in *Cxcr2*^−/−^ mice (**A**) Weight of placebo and IL-1Ra treated *Cxcr2*^−/−^ mice during the course of infection. (**B**) Fungal burden measured by the luminescence signal by *in vivo* conversion of luciferin by the luciferase expressing *A. fumigatus* (strain 2/7/1) at day 1, 2 and 3 p.i. Data is represented as scatterplot and median and were compared for significance using the Mann-Whitney U test. (C) Pro-inflammatory mediators CXCL1, IL-1α, IL-1β, IL-6, and G-CSF measured in lung homogenates at day 3 p.i. in placebo and IL-1Ra treated *Cxcr2*^−/−^ mice, and are represented as mean ± SEM and were compared for significance using the Mann-Whitney U test. (D) Hypoxia measured by the fluorescent HypoxiSense 680 probe (Perkin Elmer) at day 3 p.i., and is shown as scatterplot and medians were compared for significance using the Mann-Whitney U test.

Since IL-1Ra treatment partially reduced hypoxia and mortality in cortisone-acetate immunosuppressed mice, we investigated whether IL-1Ra treatment could also be beneficial in the genetically susceptible *Cxcr2*^−/−^ mice. Compared to placebo treated *Cxcr2*^−/−^ mice, IL-1Ra-treated *Cxcr2*^−/−^ mice did not show differences in fungal burden during the 3 day course of infection and no differences in weight loss could be observed between both groups (Fig. 6A,B). No significant differences in levels of inflammatory cytokines could be detected in the lungs (Fig. 6C). Although the IL-1Ra- treated *Cxcr2*^−/−^ mice demonstrated more variation in their hypoxia levels compared to placebo treated *Cxcr2*^−/−^ mice, no trend towards reduced or increased hypoxia could be observed between both groups (Fig. 6D).

## Discussion

Invasive fungal growth and cellular injury are two principal stimuli that trigger inflammation during IPA. The damage caused to the pulmonary tissue by the excessive inflammatory responses and invasive growth of the fungus results in hypoxia, generating a hypoxic microenvironment in which the fungus can adapt and proliferate^8^,^11^,^12^,^13^,^15^. In the current study the interaction between hypoxia and inflammation was investigated in two distinct murine models of IPA. *Cxcr2*^−/−^ mice incapable of efficiently recruiting neutrophils^23^,^24^ and cortisone-acetate immunosuppressed mice were used to study this interaction. We observed that mice with an attenuated host response, either due to *Cxcr2* deficiency or due to corticosteroid immune suppression, were more susceptible to aspergillosis and the infection resulted in severe pulmonary inflammation. This increased inflammation correlated with development of pulmonary hypoxia. Among the inflammatory cytokines measured in the lung, we found that the IL-1 cytokines (IL-1α and IL-1β) were present in high levels. Previously, an interaction between IL-1 signalling and hypoxia via HIF1α has been reported in multiple studies^30^,^31^,^32^,^33^. Therefore we investigated whether blockade of the IL-1R could reduce pulmonary hypoxia during IPA. Our data suggest that targeting IL-1 signalling could be beneficial in corticosteroid-immunosuppressed mice by reducing hypoxia and leading to improved outcome of IPA.

Using live imaging and assessment of expression of hypoxia-inducible genes we were able to detect and quantify hypoxia-related changes through the expression of carbonic anhydrase (CAIX) and the genes *Pgk1, Glut1, Vegfa, Hprt, Nos2, Car9* (CAIX) and *Hif1α* within the lungs of mice suffering from IPA. Under hypoxic conditions, HIF-1α becomes stabilized and translocates to the nucleus, where it heterodimerizes with the constitutively expressed HIF-1β. This heterodimer subsequently binds to hypoxia-response element sites in genes required for adaptation to hypoxia, including CAIX. CAIX is a transmembrane cell surface enzyme which catalyses the reversible interconversion of CO_2_ to bicarbonate and a proton. CAIX is an important biomarker in the study of hypoxia. It is overexpressed in response to tumor hypoxia in many common tumor types^38^.

A number of studies have employed radio- or fluorescent-labeled CAIX antibodies^39^,^40^,^41^ and CAIX inhibitors^42^ for the detection or characterization of hypoxia-associated CAIX expression. However, the majority of these previous studies relied on *ex-vivo* evaluation of tumor tissues or limited 2D fluorescence imaging *in vivo*. In the present study, we aimed to establish HS680 as a non-invasive *in vivo* imaging agent for selective and quantitative imaging of CA IX expression as a marker of lung hypoxia in the context of fungal infection.

Using the fluorescent hypoxia marker HS 680 together with our bioluminescent strain of *A. fumigatus*, we were able to compare the fungal burden in relation to degree of hypoxia during the first three days of infection in the lungs as well in the sinus areas. It must be noted, however, that pulmonary hypoxia can bias the luminescence towards indicating a lower fungal burden, since the firefly luciferase luminescence signal is oxygen dependent^6^,^27^. To control for this possible limitation fungal burden was additionally assessed in lung sections. In the *Cxcr2* knock out mice, the fungal burden measured by luminescence was not significantly different from the WT mice at day 3 p.i. However, lung sections show foci with massive infiltration of hyphae. Along with this, the hypoxia signal was significantly higher in the *Cxcr2* mice. In the cortisone-acetate immunosuppression model, immunosuppressed mice showed lung foci infiltrated with hyphae in relation to a higher luminescent signal associated to severe hypoxia.

One important aspect to consider during IPA is to what extent the excessive inflammation and the tissue injury-inducing hypoxia is detrimental to the host. In fact, hypoxia is a condition that closely resembles inflammation; it induces the expression of inflammatory cytokines^43^,^44^. Hypoxia in aspergillosis has been observed previously in various murine models of aspergillosis. While investigating the role of immune effector cells in the host defence against *A. fumigatus*, we previously demonstrated that at D3 day post-infection, lungs from cortisone acetate-treated mice displayed severe tissue necrosis and hypoxia^6^. Other studies using immunodeficient mice^7^,^8^ with IPA, also observed severe hypoxia at the site of infection where *A. fumigatus* hyphae are invasively growing in the pulmonary tissue. Also in a murine cystic fibrosis aspergillosis model, severe hypoxia was observed and was linked to the RAGE receptor^17^. However, none of these studies attempted to reduce hypoxia. This is to our knowledge the first study that was able to reduce hypoxia during IPA by targeting the inflammatory response, more specifically, the IL-1 pathway. In line with our study, hypoxic epithelial cell necrosis in a murine model of cystic fibrosis without *Aspergillus* infection showed that airway neutrophilia and mortality could be reduced by IL-1Ra treatment^36^.

By targeting the IL-1 axis we were able to reduce pulmonary hypoxia assessed by CAIX activity and hypoxia induced gene expression and improve modestly but significantly the outcome in the corticosteroid immunosuppression model of IPA. However, in the CXCR2 model, hypoxia could not be reduced by IL-1Ra treatment. One crucial difference between both models is that in the *Cxcr2*^−/−^ mice the neutrophil recruitment via one of the CXC chemokines family is inefficient due to absence of the CXCR2 receptor^24^, while in the corticosteroid immunosuppression model there is no direct inhibition of neutrophil recruitment^5^. However neutrophil effector functions in eradicating the fungus are reduced by corticosteroids^5^. Therefore, neutrophils could be major contributors to tissue damage inducing hypoxia in this corticosteroid model, as previously suggested^6^,^7^. In contrast, the *Cxcr2*^−/−^ model, where neutrophils are inefficiently recruited resulting in massive outgrowth of the conidia that leads to tissue damage and hypoxia. Inhibiting IL-1 signalling will reduce neutrophil recruitment to the lungs^45^,^46^ and in this way reduce collateral damage to the pulmonary tissue, which is mediated by the neutrophils. Therefore, we suggest that the differences between both models is caused by the difference in the underlying mechanisms that leads to tissue injury and hypoxia, with the *Cxcr2*^−/−^ model displaying a lack of neutrophils leading to increased fungal burden and tissue damage, and detrimental neutrophil responses in the corticosteroid model leading to hypoxia and tissue damage which is amendable to IL-1Ra treatment.

An important aspect to notice is that host immune cells need to be able to adequately sense hypoxia by HIF1α to be able to clear *Aspergillus* as was recently illustrated by an increased susceptibility to aspergillosis when myeloid cells lack HIF1α^47^. This shows the important balance of sensing of hypoxia and having adequate responses to tissue damage caused by invasive aspergillosis. These balanced immune responses are further highlighted by the role of IL-1 in invasive aspergillosis. IL-1R signalling^29^, and in particular IL-1α^28^, plays a crucial role in the activation of the initial host defence against *Aspergillus* by mediating early neutrophil recruitment in the first 24 hours of infection. Treatment with an inhibitor of IL-1 signalling is therefore difficult because there is a risk of compromising early host defence that is crucial for the host defence to gain the upper hand and clear invading fungi. The observation that IL-1 signalling is crucial for early host defence^28^ was used to determine the time point to initiate recombinant IL-1Ra treatment. We allowed IL-1 signalling for the first 24 hours of infection, after which IL-1 signalling was inhibited with recombinant IL-1Ra, Anakinra, which resulted in reduced hypoxia and an improved outcome and disease course. However, it remains difficult to determine whether the time point for initiation of IL-1Ra treatment was optimal. Significant defects of neutrophil recruitment to the lungs in IL-1R^−/−^ have been observed as early as 10-hour p.i^29^, it could therefore be that the protective role of IL-1 is very early during infection. Nevertheless, the possibility of an immunomodulatory treatment to reduce IL-1 mediated immunopathology could open new treatment options that can be used in the clinics for patients where inflammation has gained the upper hand rather than defective fungal clearance. Severe inflammation is a key player in determining outcome of aspergillosis when the host is relatively immunocompetent, such as in CGD^35^,^48^,^49^,^50^,^51^,^52^, CF^17^,^36^,^53^, and ABPA^53^,^54^,^55^,^56^,^57^. One important aspect is that in our model, only the inflammatory axis was targeted, while no antifungal therapy was initiated. A combination of antifungal therapy with immunomodulatory strategies is believed to be a promising avenue to improve outcome of invasive fungal disease^57^,^58^,^59^,^60^,^61^,^62^. Our preliminary data suggest that targeting IL-1 signalling in combination with antifungal treatment has an increased potential to improve outcome of aspergillosis. Yet it remains difficult to determine which patients will benefit from adjunctive immunotherapy, therefore critical assessment of cases is required to fit a tailored treatment strategy.

In summary, our data demonstrates that inhibiting pro-inflammatory IL-1 signaling with recombinant IL-1Ra can reduce hypoxia and improve the outcome of IPA in corticosteroid immunosuppressed mice. We therefore suggest that targeting IL-1-dependent inflammatory responses to reduce detrimental hypoxia-driven inflammation might prove as a promising immunomodulatory strategy in addition to regular antifungal treatment to improve outcome of invasive aspergillosis.

## Methods

### Strain culturing and mouse infection

For all experiments the *A. fumigatus* luminescent strain 2/7/1 containing the codon-optimized *Photinus pyralis* luciferase gene *luc*_Opt_ was used^63^. This bioluminescent *A. fumigatus* strain was cultured on 2% malt extract agar slants for 8 days at room temperature. Conidia were harvested by scrapping them from the slant culture with 2 ml of phosphate buffered saline supplemented with 0.1% Tween 20 (PBST). The suspension was filtered through a 40 μm cell strainer (BD Falcon, Bedford MA, USA) to separate conidia from contaminating mycelium.

### Mice and Ethics statement

Throughout this study male BALB/cJ mice (23 to 28 g, 8 weeks old) were used and supplied by the breeding center R. Janvier (Le Genest Saint-Isle, France). All procedures were carried out in accordance with Pasteur Institute guidelines in compliance with European animal welfare guidelines. This study was approved by the ethical committee for animal experimentation CETEA (Comité d’éthique en experimentation animale, Project license number 2013–0020). Upon arrival, animals were placed in isolated ventilated cages in groups of five. Intranasal infection was performed in (i) Cortisone-acetate immunosuppressed mice. Cortisone acetate was suspended in sterile phosphate buffered saline (PBS) to give a final concentration of 125 mg/mL. The suspension was sonicated at 37 °C for at least 30 minutes to prepare a homogenous suspension. Immunosuppression was performed as described previously^64^, whereby mice were immunosuppressed with two single doses of 25 mg cortisone acetate (Sigma Aldrich, St Louis, MO), which were injected intraperitoneally three days before and immediately prior to infection with conidia (day 0).

At the day of infection, mice were anesthetized by intramuscular injection of 150 μL of a solution containing 10 μg/mL of ketamine and 10 μg/mL xylazine and inoculated intranasally with a dose of either 5 × 10^5^ or 2 × 10^5^ conidia in 25 μL of PBS. (ii) *Cxcr2* knock out mice. In comparison to the pharmacologic immunosuppressive treatment, we used mice deficient in the chemokine receptor CXCR2 (*Cxcr2*^−/−^ mice). These mice display a defect in neutrophil recruitment and are susceptibile to *A. fumigatus*^6^,^23^,^65^. The susceptibility of these mice was compared to BALB/c wild type mice following inoculation with 5 × 10^7^ conidia in 25 μL of PBS.

### Measurement of hypoxia using HypoxiSense 680 probe

Several invasive and non-invasive approaches have been developed to measure tumor oxygenation including hypoxia bio-marker targeted agents with labels that can be detected by imaging techniques. There has been considerable interest in fluorescent optical reporters for CAIX expression. Among these agents, it has been reported that HS680 has high *in vitro* and *in vivo* specificity for CAIX. It accumulates preferentially in hypoxic regions of CAIX positive tumors and can be used to non-invasively detect and quantify CAIX^25^.

Pairing HypoxiSense with optical fluorescent imaging technology allows for the first time to image and quantitate tumor sub-regions undergoing hypoxia-related changes, non-invasively and *in vivo*. In our settings, each mice received intravenously at day 2 p.i 100 μL of the fluorescent imaging HypoxiSense 680 (2 nmol) from Perkin Elmer.

## *In vivo* imaging

### (i) Bioluminescence

Images were acquired using an IVIS 100 and spectrum systems (Perkin Elmer) according to the manufacturer’s instructions and as previously described^66^. In brief, 100 μL of PBS containing 3.33 mg D-luciferin was intraperitoneally injected in mice before each measurement. Mice were anesthetized using a constant flow of 2.5% isofluorane mixed with oxygen using an XGI-8 gas anesthesia system (Xenogen Corporation). Images from mice were acquired 10 min after luciferin injection. Acquisition and quantification were performed using Living Image software version 3.1 (Xenogen Corporation). Quantification of photons per second emitted by either the lungs or sinus areas was performed by defining regions of interest.

### (ii) Fluorescent tomography (FMT 100 Perkin Elmer)

Infected mice were anesthetized by isoflurane/oxygen mixture gas anesthesia system. Experimental and control mice were then imaged using fluorescence tomography (FMT 2500^LX^ Pre-Clinical Imaging System, PerkinElmer, Boston, MA PerkinElmer, Boston, MA). Briefly, the anesthetized mice were positioned in the imaging cassette which was then placed into the FMT imaging chamber. A NIR laser diode trans illuminated (i.e. passed light through the body of the animal to be collected on the opposite side) the infected regions, with signal detection occurring via a thermoelectrically cooled CCD camera placed on the opposite side of the imaged animal. Appropriate optical filters allowed collection of bothfluorescence and excitation datasets, the entire image acquisition sequence taking approximately 5–6 min per mouse. The collected fluorescence data was reconstructed (TrueQuant™ software, PerkinElmer, Boston, MA) for the quantification of three-dimensional fluorescence signal within the tumors. Three-dimensional regions of interest (ROI) were drawn in the lung or sinus areas. For visualization and analysis purposes, TrueQuant software provided three dimensional (3D) images and quantification in pmoles of fluorescence within ROIs. Additional technical information on FMT imaging, image re-contruction, image analysis and quantification can be found in the following review articles^67^.

These protocols enabled us to monitor and quantify for each mouse (i) fungal growth by bioluminescence and (ii) hypoxia by the cell surface expression of carbonic anhydrase (CAIX).

### Bronchoalveolar lavages

Bronchoalveolar lavage (BAL) fluid was harvested as previously described^68^. Mice were euthanized by injection of Pentobarbital (Sanofi Santé Animale, Libourne, France) and the respiratory tract was exposed by dissection. A small incision was made near the top of the trachea, and a blunt-end 20-gauge needle was inserted and tied in place with surgical thread around the trachea. BAL fluid was obtained by 10 rounds of filling the lungs with 0.7 mL PBS and withdrawing as much of the liquid as possible. The samples were centrifuged to collect BAL fluid cells. BAL fluid cells were washed and resuspended in 1 mL PBS.

### Histopathology

Mice were euthanized at day 3 p.i. Lungs were immediately fixed in 4% neutral-buffered formalin and embedded in paraffin. Five μm sections were cut and stained with Grocott’s methenamine silver staining (GMS) for detection of fungi^69^.

### Gene expression

Mouse lungs were homogenized in 500 μL TRIzol reagent (Invitrogen) and subsequently RNA was isolated according to the protocol supplied by the manufacturer. RNA was reverse transcribed into cDNA using iScript cDNA synthesis kit (Biorad, Hercules Ca). Quantitative real-time PCR (qPCR) was performed using power SYBR Green PCR master mix (Applied Biosystems, Carlsbad, CA) and following primers: *Gapdh* Fwd 5′-AGGTCGGTGTGAACGGATTTG-3′ and Rev 5′-TGTAGACCATGTAGTTGAGGTCA-3′, *Pgk1* Fwd 5′-CAAATTTGATGAGAATGCCAAGACT-3′ and Rev 5′-TTCTTGCTGCTCTCAGTACCACA-3′^70^, *Glut1* Fwd 5′-GGGCATGTGCTTCCAGTATGT-3′ and Rev 5′-ACGAGGAGCACCGTGAAGAT-3′^70^, *Vegfa* Fwd 5′-AGTCCCATGAAGTGATCAAGTTCA-3′ and Rev 5′-ATC CGCATGATCTGCATGG-3′^70^, *Hprt* Fwd 5′-TTATCAGACTGAAGAGCTACT-3′ and Rev 5′-TTACCAGTGTCAATTATATCTTCAACAATC-3′^70^, *Hif1α* Fwd 5′-ACCTTCATCGGAAACTCCAAAG-3′ and Rev 5′-ACTGTTAGGCTCAGGTGAACT-3′,, Car9 (CAIX) Fwd 5′- CCGGAACTGAGCCTATCCAAC-3′ and Rev 5′- GCAAGGCCCGGTATTCCTG-3′, *Il1b* Fwd 5′-GCAACTGTTCCTGAACTCAACT-3′ and Rev 5′-ATCTTTTGGGGTCCGTCAACT-3′, *Nos2* Fwd 5′-CGTTTCGGGATCTGAATGTGA-3′ and Rev 5′-GGGCAGCCTGTGAGACCTT-3′, and *Mpo* Fwd 5′-GTCAGCTGTAACACCCTTCCTAAAC-3′ and Rev 5′-CCGGCAGACTCCCAACCT-3′. PCR was performed using an Applied Biosystem StepOne PCR system using PCR conditions 2 min 50 °C, 10 min 95 °C followed by 40 cycles at 95 °C for 15 sec and 60 °C for 1 min. The RNA expression of genes of interest were corrected for differences in loading concentration using the signal of housekeeping protein GAPDH. Subsequently, the expression of immunocompetent mice was set as 1+/− SEM to measure changes relative expression in the cortisone acetate immunosuppressed mice and cortisone acetate immunosuppressed mice treated with IL-1Ra.

### Flow Cytometry

Lung samples were processed to single cell suspension by use of commercial digestion kit (Milteny Biotec) according to manufacturer’s instructions. For surface staining cells from BAL and lungs were incubated for 30 minutes with appropriate amounts of antibodies in the presence of Fc block (BD Bioscience), after which cells were washed and stained with viability dye (eBioscience). Antibodies used were directed against mouse CD3 (145-2CII) and CD19 (ID3), NKp46 (29A1.4), F4/80 (BM8), Gr-1 (RB6-8C5), CD11c (HL3), CD11b (M1/70), Ly6C (HK1.4), Ly6G (1A8-Ly6g) were purchased from Biolegend, BD Biosciences and eBioscience. Flow cytometric data was acquired on a MACSQuant device (Miltenyi Biotec) and analysed using FlowJo software (TreeStar).

### Enzyme-Linked Immunosorbent assay (ELISA)

Lungs homogenates were obtained following disruption in saline using the Retsch Mixer Mill 301 homogenizer. CXCL1, IL-1α, IL-1β, IL-6 and G-CSF, concentrations were determined in lungs supernatants by ELISA as specified by the manufacturer (DuoSet; R&D Systems).

### Statistical analysis

Comparison of luminescence, hypoxia and inflammation within the different groups of mice was performed using two way ANOVA followed by Bonferoni post test. Differences in cytokine levels between experimental groups were assessed using the Mann-Whitney U test.

All tests were performed using Graph pad Prism software. *p < 0.05; **p < 0.005; ***p < 0.001; ****p < 0.0001.

## Additional Information

**How to cite this article**: Gresnigt, M. S. *et al*. Reducing hypoxia and inflammation during invasive pulmonary aspergillosis by targeting the Interleukin-1 receptor. *Sci. Rep.* **6**, 26490; doi: 10.1038/srep26490 (2016).

## Supplementary Material

Supplementary Information
