# Supplementary material for: Reducing hypoxia and inflammation during invasive pulmonary aspergillosis by targeting the Interleukin-1 receptor
Source: Sci Rep. 2016 May 24;6:26490. doi: 10.1038/srep26490 (PMC4877709; doi:10.1038/srep26490)

Reducing hypoxia and inflammation during invasive pulmonary aspergillosis by targeting the Interleukin-1 receptor

Mark S. Gresnigt[1,2], Abdessalem Rekiki[3], Orhan Rasid[1], Amélie Savers[4], Grégory Jouvion[5], Eric Dannaoui[6], Marianna Parlato[7], Catherine Fitting[1], Matthias Brock[4], Jean-Marc Cavaillon [1], Frank L. van de Veerdonk[2] and Oumaïma Ibrahim-Granet[1]*

Supplementary information

Figure S1 Survival following IL-1Ra (Anakinra)/caspofungin combination treatment

Cortisone-acetate treated mice were challenged with 2 × 10^5 conidia and treated either with caspofungin (10mg/kg/d) or with caspofungin (10mg/kg/d) and Anakinra (10mg/kg/d) for 10 days. Mice were daily monitored for survival for 15 days.

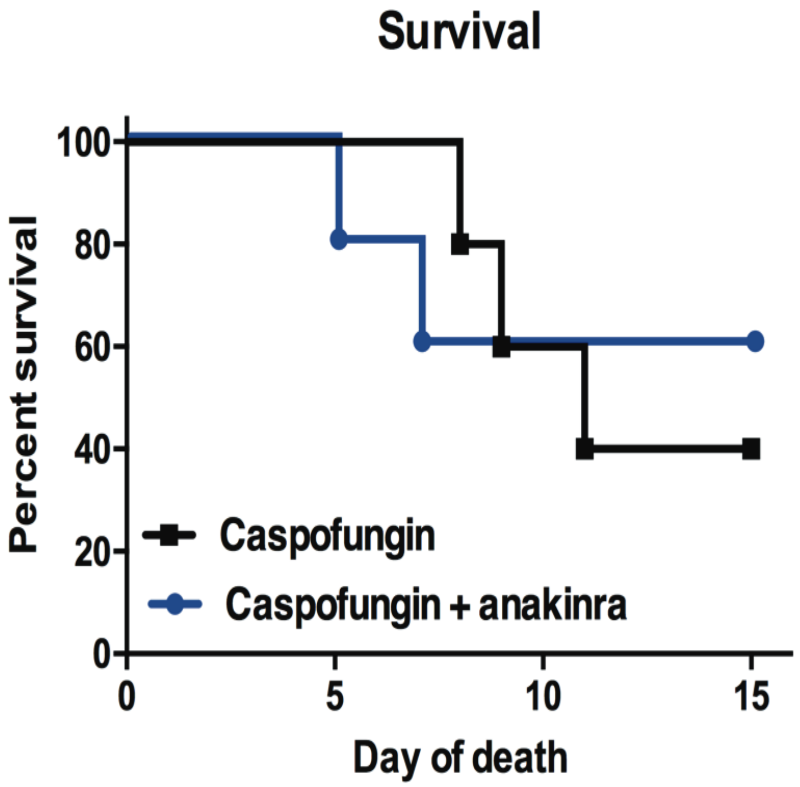

Supplement: Supplementary Information [file srep26490-s1.pdf]
